# Associations between HIFs and tumor immune checkpoints: mechanism and therapy

**DOI:** 10.1007/s12672-023-00836-7

**Published:** 2024-01-02

**Authors:** Jiayu Liu, Ying Jiang, Lingyan Chen, Zhiwen Qian, Yan Zhang

**Affiliations:** 1 Department of Oncology, Wuxi Maternal and Child Health Hospital, Wuxi School of Medicine, Jiangnan University, Wuxi, 214002 Jiangsu China; 2 Wuxi Maternal and Child Health Hospital, Nanjing Medical University, Nanjing, 214000 Jiangsu China

**Keywords:** HIFs, Mechanism, Immune checkpoints, Immunotherapy, Combination therapy

## Abstract

Hypoxia, which activates a variety of signaling pathways to enhance tumor cell growth and metabolism, is among the primary features of tumor cells. Hypoxia-inducible factors (HIFs) have a substantial impact on a variety of facets of tumor biology, such as epithelial-mesenchymal transition, metabolic reprogramming, angiogenesis, and improved radiation resistance. HIFs induce hypoxia-adaptive responses in tumor cells. Many academics have presented preclinical and clinical research targeting HIFs in tumor therapy, highlighting the potential applicability of targeted HIFs. In recent years, the discovery of numerous pharmacological drugs targeting the regulatory mechanisms of HIFs has garnered substantial attention. Additionally, HIF inhibitors have attained positive results when used in conjunction with traditional oncology radiation and/or chemotherapy, as well as with the very promising addition of tumor immunotherapy. Immune checkpoint inhibitors (CPIs), which are employed in a range of cancer treatments over the past decades, are essential in tumor immunotherapy. Nevertheless, the use of immunotherapy has been severely hampered by tumor resistance and treatment-related toxicity. According to research, HIF inhibitors paired with CPIs may be game changers for multiple malignancies, decreasing malignant cell plasticity and cancer therapy resistance, among other things, and opening up substantial new pathways for immunotherapy drug development. The structure, activation mechanisms, and pharmacological sites of action of the HIF family are briefly reviewed in this work. This review further explores the interactions between HIF inhibitors and other tumor immunotherapy components and covers the potential clinical use of HIF inhibitors in combination with CPIs.

## Introduction

The majority of solid tumors lack adequate oxygenation areas and are hypoxic (pO_2_ pressure < 8 mmHg) [1]. Low oxygen (O_2_) supply caused by aberrant vascularization and excessive O_2_ demand by tumor cells, which show remarkably increased proliferation and aggravated metabolic activity, are the causes of tumor hypoxia [2]. The biological mechanism known as the hypoxic adaptation response, which is necessary for the survival of cells under hypoxia conditions, involves the stimulation of many molecular signaling pathways that enhance erythropoietin synthesis, angiogenesis, and metabolic reprogramming to promote glycolysis [3]. The activity of transcription factors (TFs) known as hypoxia-induced demand of cells stimulates a variety of signaling pathways necessary for the survival of cells, primarily the TF hypoxia-inducible factors (HIFs), which determines the activation of the hypoxic adaptive response [4, 5].

The onset and metastasis of cancer are significantly influenced by tumor-associated hypoxia. HIFs perform an integral function in the adaptation of tumor cells to hypoxia by enhancing the oncogene transcription and negatively regulating the transcription of the suppressor gene [6]. HIFs are important in a variety of fundamental elements of cancer biology such as angiogenesis [7, 8], maintenance of stem cells [9–11], reprogramming of energy metabolism [12, 13], signaling of autocrine growth factors [14, 15], epithelial-mesenchymal transition (EMT) [16–18], invasion [19], metastasis [20, 21], and resistance to radiotherapy [22] and chemotherapy [23]. Numerous studies and clinical findings have demonstrated that HIFs are potent targets for the therapy of cancer. The development of tumors, vascularization, and metastasis are initially associated with HIF-1a or HIF-2a levels in both experimental animals and human therapeutic trials. Furthermore, HIF activity is increased by the gain-of-function of oncogenes and viral transforming genes as well as the loss-of-function of genes that inhibit cancers, particularly Von Hippel-Lindau (VHL) genes [24]. Additionally, recent advancements in multi-omics techniques (metabolomics, proteomics, transcriptomics, and genomics) and experimental cancer metabolism modeling have provided new information on the molecular mechanisms of HIFs-deficient cancer cells undergoing hypoxia. A growing variety of pharmacologic treatments have been shown to suppress HIF activity and prevent the growth of tumor xenografts via various molecular mechanisms. The development of pharmacological drugs to modify the HIF signaling system has lately sparked considerable attention. In preclinical and clinical contexts, a variety of methods targeting malignant cells caused by hypoxia are currently being studied.

Immune checkpoint inhibitors (CPIs) play an obvious function in immunotherapy. The advantages of combining numerous CPIs have recently provided novel insight into how to resolve ongoing adverse immunological events. The growing body of research pointing to the potential benefits of combining HIF inhibitors with CPIs for enhancing antitumor immune responses and reducing malignant cell plasticity and treatment resistance will be examined in this review.

## Structure of HIFs

Human tissues have three distinct HIFs, HIF-1, HIF-2, and HIF-3, which are strictly modulated by alterations in oxygen tension [25]. While HIF-3's function is less understood, HIF-1 and HIF-2 are transcriptional modulators with both distinct and overlapped target genes. The expression of HIF-2 and HIF-3 in the human endothelium starts with chronic hypoxia, whereas HIF-1 controls the acute response to hypoxia. Unlike HIF-2α and HIF-3α, which are only expressed in certain tissues, HIF-1α, a 120 kDa oxygen-sensitive subunit ubiquitously, is expressed in all tissues.

**Fig. 1:**
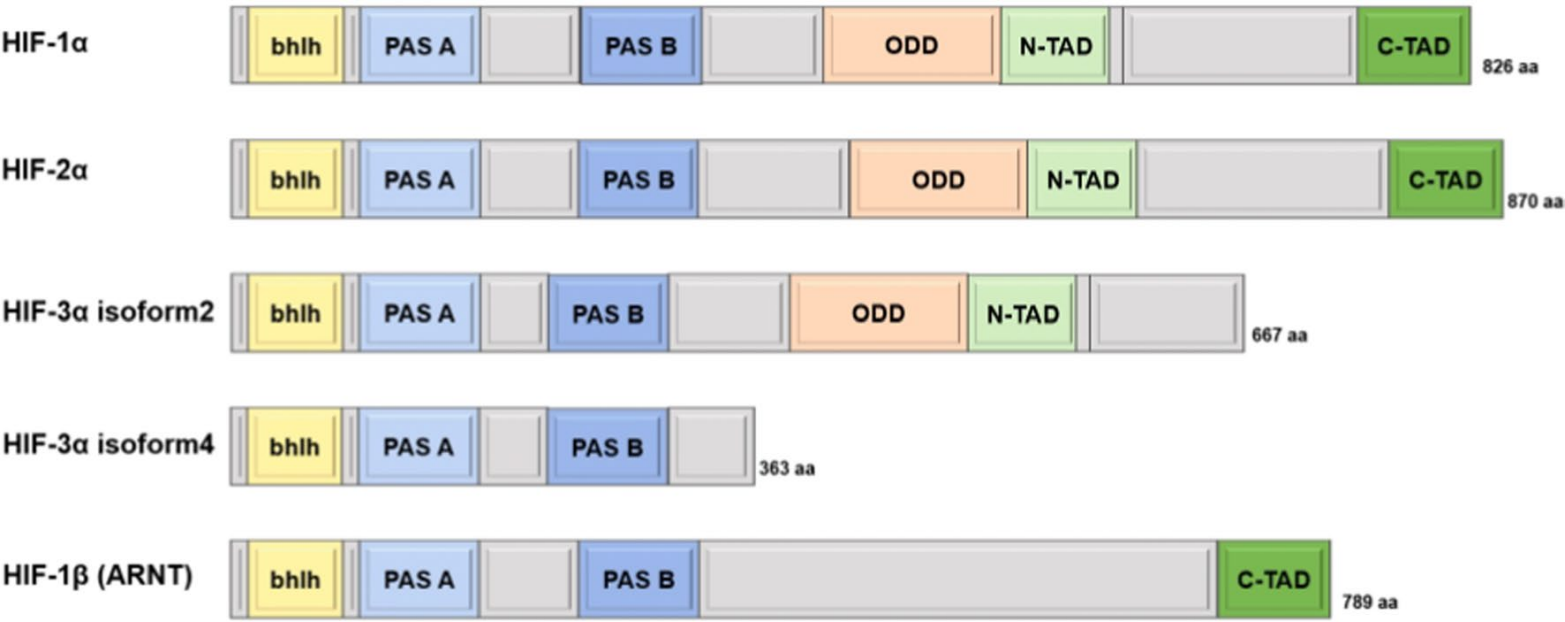
Schematic representation of the HIF subunit domain structures. *bHLH* basic helix–loop–helix, *PAS* PER–ARNT–SIM, *ODD* oxygen-dependent degradation domain, *N/C-TAD* N/C-terminal transactivation domain, *aa* number of amino acid residues

HIFs are composed of heterodimers and the subunits α and β. Hypoxia induces the production of the HIF-1/2/3α alpha subunits, which are found in cell membranes. In the nucleus, beta subunits (aryl hydrocarbon receptor nuclear translocator) HIF-1β/aryl hydrocarbon receptor nuclear translocator (ARNT), HIF-2β (ARNT2), and HIF-3β (ARNTL), respectively, are expressed constitutively [26]. The PER-ARNT-SIM(PAS) and basic helix-loop-helix (bHLH) motifs that facilitate heterodimerization, as well as DNA binding, are located at the amino-terminal end of both the α and β subunits [27]. Two transactivation domains (TADs) (i.e., N-TAD and C-TAD) and oxygen-dependent degradation domain (ODD) form the carboxy-terminal motif of HIF-1/2α, which controls both the proteins’ transcriptional activity and stability, respectively, [27]. Additionally, nuclear localization signals C-NLS and N-NLS, correspondingly on the C- and N-termini of the α subunits point them toward the nucleus [28]. The N-TAD domain is the aspect where HIF-1α and HIF-2α differ the most from one another, sharing 48% of their amino acid sequence identity [29]. HIF-3α shares bHLH and PAS motifs with HIF-1/2α although it does not have the C-terminal transactivation motif [30]. Nonetheless, alternative splicing (AS) of HIF-3α and the use of various promoters lead to at least four distinct HIF-3α mRNA variants that encode for ≥ isoforms [31]. The inhibitory PAS motif protein, a shortened protein that blocks HIF-1/2 function in cell culture, is the HIF-3 variant that has received the most research to date [32]. Conversely, it was discovered that the other human HIF-3 variants upregulated gene levels, proving that HIF-3 is also a crucial transcriptional modulator of hypoxia signaling [33, 34]. The HIF subunit domains are shown in Fig. 1.

## HIF stability modulation

The stability of all three HIF-α proteins is regulated by oxygen [26]. Factor-inhibiting hypoxia-inducible factor-1 (FIH-1), Prolinehydroxylases (PHDs), and hydroxylase enzymes hydroxylate alpha subunits posttranslationally under normoxia. Irrespective of oxygen tension, HIF-1α is often inactivated in healthy cells yet commonly maintained in cancerous cells [35, 36]. A crucial step that triggers the expression of numerous genes implicated in diverse biological processes is the transportation of stable HIF-1α to the nucleus as well as its interaction with HIF-1β ARNT [24]. These hydroxylase enzymes aim to polyubiquitinate and degrade the alpha subunits under normoxia [37]. Specialized proline residues within ODD domains are subjected to hydroxylation reliant on PHD, which recruits the VHL tumour suppressor protein (pVHL) as well as other protein cofactors and causes the 26S proteasome to degrade the alpha subunits. PHDs need iron ions (Fe^2+^), ascorbic acid, 2-oxoglutarate, and molecular oxygen to hydroxylate HIF-α [38]. Additionally, various PHD isoforms have varied HIF specificities. For example, PHD-2 activity is mostly HIF-1α-specific [39], while PHD-3 controls HIF-2α levels primarily [40]. The heterodimeric complex's transcriptional activity is regulated by the second hydroxylase, FIH-, by hydroxylating one asparagine residue in the transactivation motifs of HIF-1/2 [41]. Such posttranslational modification precludes the dependent mobilization of the co-activators CREB-binding protein (CBP, sometimes referred to as CREBBP) and p300, which exhibit histone acetyltransferase activity, serving as a late-stage key phase in the HIF activation process [42]. Molecular oxygen is also necessary for the FIH-1 action. It's worth noting that FIH-1 selectively hydroxylates HIF-1α and needs less oxygen tension to stay active than PHD-2 [43].

**Fig. 2:**
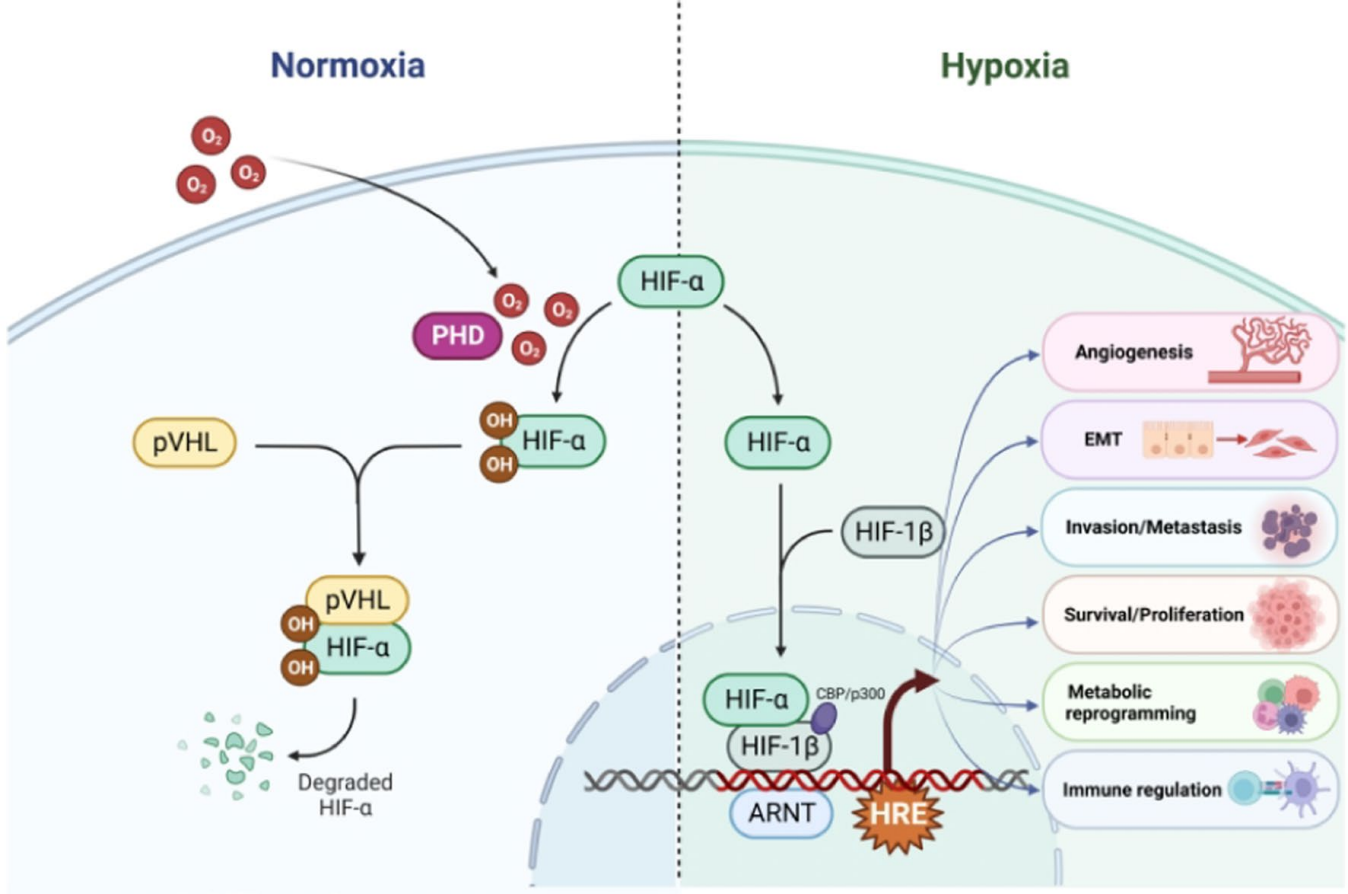
Modulation of the transcription factors in the HIF family

Because the hydroxylases in hypoxia lack an oxygen substrate, HIF-1α accumulates, moves to the nucleus, and afterward forms complexes with HIF-1β, its co-factor. In normoxia, HIF protein content and transcriptional activity are kept low by the action of both PHD-2 and FIH-1 [44]. Low oxygen tension, on the other hand, reduces the activity of PHD-2 and FIH-1 and stabilizes the HIF-alpha subunit. Following translocation to the nucleus, the alpha subunits dimerize with the beta subunits for the purpose of generating HIF complexes that are transcriptionally active [45]. By attaching to the hypoxia response element (HRE) sequences within promoters of their distinct and shared target genes, HIF-1/2 facilitates the endothelium hypoxic response and upregulates those genes [30]. Numerous genes, such as those involved in cell proliferation [46], metastasis [20, 47–49], glycolysis [50, 51], pH control [52], and angiogenesis [53] are activated by the HRE, a TF-binding domain found in the promoter sequences of target genes. Despite reports of HIF-elicited negative transcriptional modulation, it nearly completely occurs indirectly [54, 55]. Lastly, under hypoxia, the Sirtuin 1 (SIRT1) induced by hypoxia preferentially deacetylates HIF-1α and enhances HIF-1 activity [56]. The control of HIF subunits in normoxia and hypoxia is depicted in Fig. 2.

HIF- subunits are hydroxylated only when Iron, oxygen, and 2-oxoglutarate are present under normoxia. The pVHL E3 ligase complex can bind to the HIF-subunit via hydroxylation, thus facilitating polyubiquitination and eventual degradation by the 26S proteasome. The PHD is inhibited in hypoxia (< 5% O2). HIF-subunits are shielded from pVHL-driven destruction and move to the nucleus after which they combine with HIF-1 to produce heterodimers. The HIF heterodimers bind to HRE present in the target genes’ DNA modulatory regions, stimulating their transcription by enlisting the transcriptional co-activators CBPP/p300, stimulating the transcription of several HIF target genes involved in tumor cell proliferation/survival, EMT, angiogenesis, metastasis/invasion, ODD metabolic reprogramming, and immunoregulation

## Specificity of HIF isoforms in the tumor immune microenvironment

A critical factor in deciding whether a tumor will progress or shrink during the course of its development and how it responds to therapy is the complex landscape known as the tumor microenvironment (TME), which is made up of immune and stromal cells. Research demonstrates the TME cells’ extraordinary heterogeneity, flexibility, and interconnectedness [57]. It was demonstrated that IL-4, IL-13, and CXCL1 suppression reduced protumorigenic myeloid-derived suppressor cells (MDSCs) and tumor-associated macrophages (TAMs) and that elevated levels of CXCL9 and CXCL10 resulted in greater infiltration of antitumor NK and CTLs cells [58]. Hypoxic stress is a key microenvironmental component that can inhibit antitumor immunity and activates many pathways that lead to the formation of resistant cancerous cells [59–61].

The TME is governed by specific microenvironmental factors, which in turn are regulated by cross-cellular communication, which produces diverse signaling outputs in different cell types. In hypoxia, for instance, hypoxia enhances the secretion of CCL28 by tumor cells in a HIF-1α-dependent mode, which enhances the recruitment of CCR10^+^ T reg cells to the tumor site, thus inhibiting the functions of cytotoxic T cells and accelerating tumor growth [62, 63]. Adenosine and the A2a receptor work together to promote immune checkpoint expression in hypoxic environments, which suppresses T cells [64]. Additionally, HIF-1α increases the expression of a protein called ADAM10 (a disintegrin and metalloproteinase domain-containing protein 10) in cancerous cells, causing major histocompatibility complex class I chain-related molecule (MICA) to be shed from a tumor cell's surface. The NKG2D activator receptor on NK and T cells is downregulated by soluble MICA, allowing cancer cells to evade [65]. Eventually, hepatocellular carcinoma (HCC) cells express ectonucleoside triphosphate diphosphohydrolase 2 (ENTPD2) when hypoxia-driven HIF-1α (but not HIF-2α) is present, which facilitates the onset and progression of syngeneic Hepa1-6 HCC tumors in mice by increasing the infiltration of MDSC into the tumor mass [66]. Overall, the HIF family of TFs controls a variety of TME activities known to modify the metabolic activity and aggressiveness of tumors as well as the environment's immunosuppressive conditions that favor tumor development. Immune checkpoint blockade (ICB) is used to accomplish the latter goal.

**Fig. 3:**
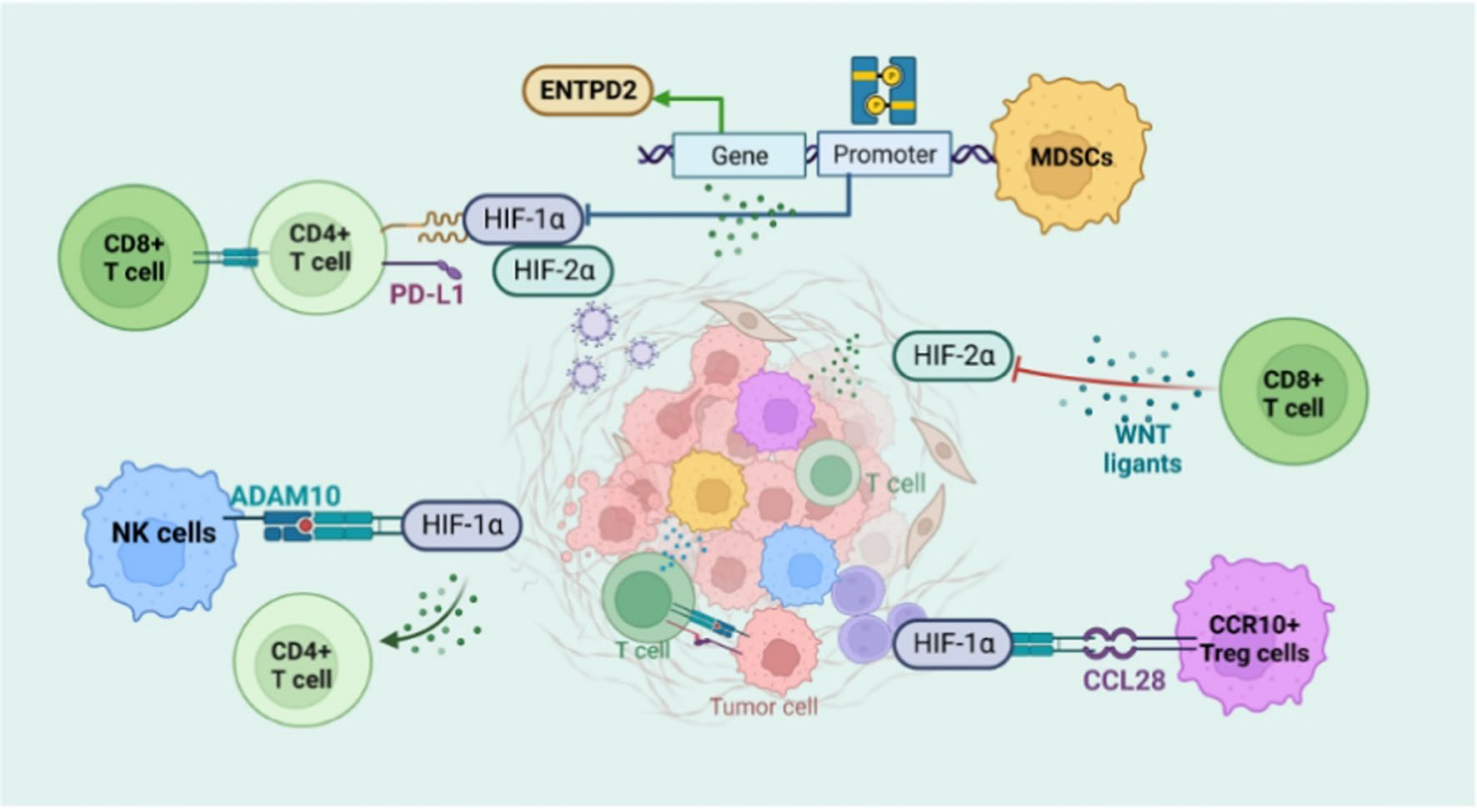
HIF transcription factors’ effects on the TME’s tumor-immune cell interaction

Additionally, there is proof that the immunological and non-immune constituents of the TME interact extensively, whereas the efficacy of adoptively transplanted tumor-specific CD8 + T cells in a syngeneic murine model of lung cancer is increased by the overexpression of superoxide dismutase (SOD3) in endothelial cells (EC) and the consequent stabilization of HIF-2α rather than HIF-1α [67]. Previous research illustrated that antisense HIF-1 and B7-1-T may enhance NK cell and CD8 T cell-elicited anticancer immune response and trigger tumor rejection by downregulating HIF-1 expression [68]. The effect of HIF transcription factors on tumor-immune cell interactions in TME is shown in Fig. 3. According to Lequeux et al., therapies that prevent HIF-1/HIF-1 dimerization can change the tumor's immunosuppressive environment into one that is permissive to NK and CD8^+^ effector T cell infiltration. These techniques may be utilized to enhance cancer immunotherapy regimens including ICB treatments and cancer vaccination in melanoma individuals who are not responding to treatment [69]. Furthermore, HIF-1 suppression and DC-based immunotherapy were confirmed to enhance survival in a breast cancer model by enhancing the proliferation and activities of cytotoxic T cells and enhancing the synthesis of type 1 interferon (IFN) [70]. Collectively, the above evidence highlights the complex and dynamic interactions between HIF-activated tumor cells and TME immune cells that affect tumor advancement, treatment response, and aggressiveness.

The production of tumor cell-specific HIF in tumor cells affects the interactions between tumor and immune cells, which control cytotoxicity and therapeutic effects induced by immune cells against tumors.

## HIF associated with immune checkpoint blockade

**Fig. 4:**
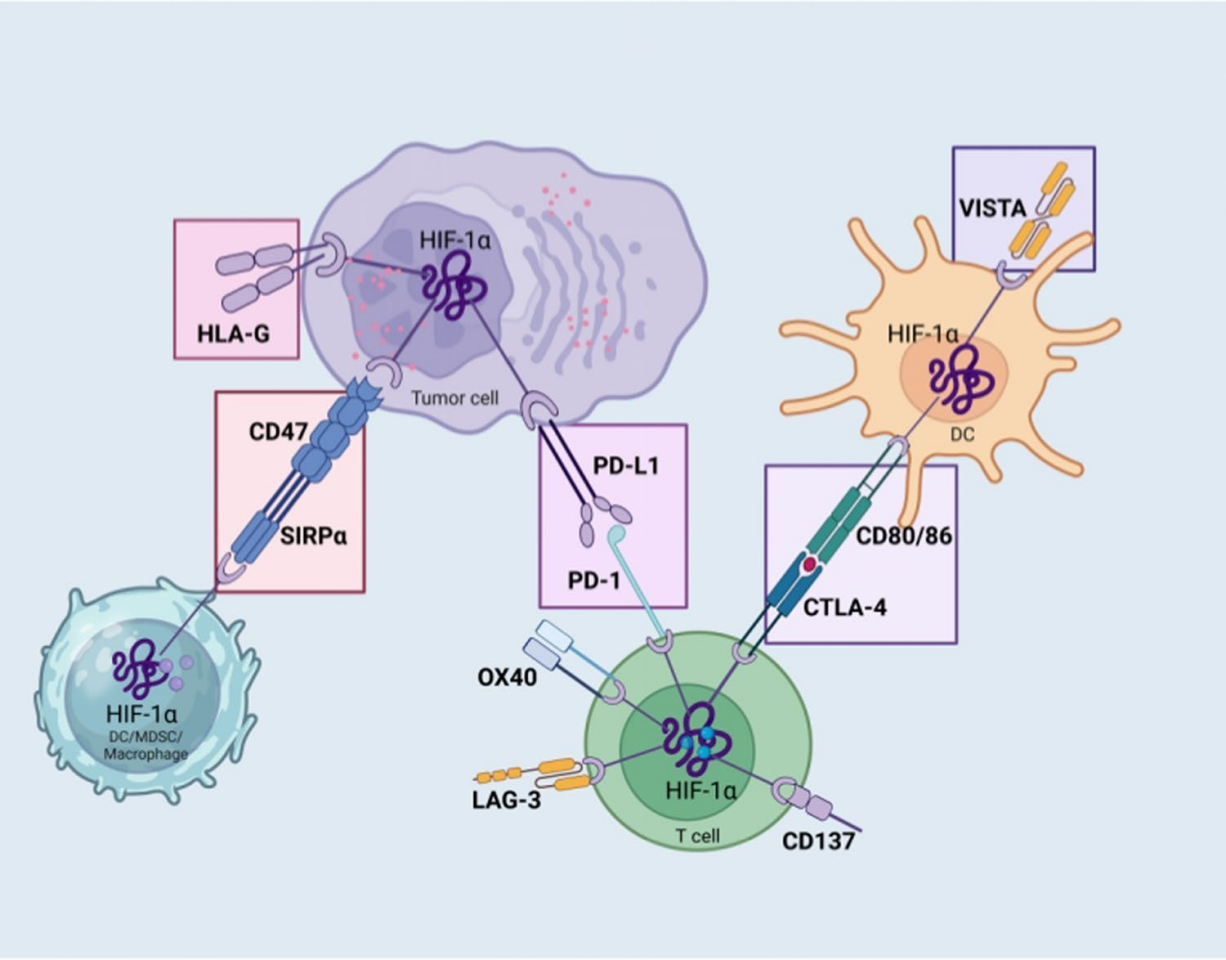
Immune checkpoint modulation in the TME under hypoxia

Hypoxia regulates multiple major immune checkpoint molecules, such as the V-domain Ig suppressor of T-cell activation (VISTA), the cluster of differentiation 47 (CD47), human leukocyte antigen G (HLA-G), and PD-L1. The interactions between HIF and these key immune checkpoints will be highlighted next (Fig. 4).

### PD-L1

By regulating the expression of the immune checkpoint PD-L1 on the MDSCs' surface, hypoxia increases the immunosuppressive characteristics of MDSCs against T cells [71–73]. Inhibiting PD-L1 under hypoxic settings encourages T cell activation driven by MDSC while attenuating IL-6 and IL-10 synthesis from MDSCs. [72]. It has been shown that hypoxia can increase the levels of PD-L1 protein in human breast and prostate carcinoma cells via HIF-1α [73]. MDSCs derived from the spleen are greatly promoted to express PD-L1 when exposed to hypoxia, according to data from mice models that bear the B16-F10 melanoma [72]. Additional research revealed that after stabilizing in hypoxic cells, HIF-1α attaches to the HRE in the PD-L1 gene’s proximal promoter [72]. Additionally, HIF-2α is also implicated in the overexpression of PD-L1 which is remarkably correlated with VHL mutation and HIF-2α stability among patients with clear cell renal cell carcinoma (ccRCC) [71]. Analysis of paraganglioma and pheochromocytoma samples revealed a substantial correlation of PD-L2 expression with carbonic anhydrase 9 (CAIX) and hypoxia-driven HIF-1α, which was not the case with PD-L1 expression [74]. These findings suggest that inhibiting HIF-1α and PD-L1/PD-L2 simultaneously might be an effective way to increase the activity of cytotoxic T cells.

### HLA-G

The immune checkpoint marker HLA-G, a non-classical MHC-I molecule, is thought to be relevant to immunotherapy [75]. There is evidence that high invasive or metastasis and negative treatment outcomes are correlated with malignant tumors' aberrant HLA-G expression [76]. Published investigations have shown that hypoxia causes HLA-G upregulation at the mRNA level [77]. In the first part of their investigation, Mouillot et al. showed that when exposed to hypoxia, HIF-1α increases the levels of HLA-G mRNA in the HLA-G–negative M8 melanoma cell lines [77]. Since the HLA-G promoter contains a small number of HREs, HIF-1 probably binds to HRE domains to activate HLA-G transcription as the mechanism driving HLA-G expression in response to hypoxia [76]. According to research by Yaghi et al., the HLA-G gene is expressed in glioma cells due to the attachment of HIF-1 to the HRE domain in exon 2; other MHC-I molecules affected by hypoxia in malignancies have received less research [78]. Furthermore, research shows that the expression of Qa-1 in mice and HLA-E in human malignant cells may both be remarkably upregulated by the combined exposure to glucose deprivation and oxygen. As a result, the cells can engage with the blocking CD94/NKG2 receptor on activated T cells and avoid being recognized by CD8^+^ T cells [79].

### CD47

The transmembrane immune checkpoint protein CD47 also referred to as integrin-associated protein, is present on the surface of both solid and hematologic tumor cells. Also, CD47 upregulation is associated with an unfavorable clinical prognosis [80]. The predominant process underlying CD47-elicited immune escape is its interplay with SIRP, which is expressed at a high level in myeloid-lineage hematopoietic cells such as MDSCs and TAMs. Through this connection, cancer cells are prevented from being phagocytosed by a strong “don’t eat me signal” delivered by SIRP phosphorylation [80]. Recent research has shown that by specifically binding to its promoter, HIF-1α may modulate the CD47 gene’s transcription. Additionally, the capacity of macrophages to phagocytose breast carcinoma cells is improved when CD47 is inhibited [81]. MDSCs and macrophages are blocked from expressing prophagocytic signaling in pancreatic adenocarcinoma due to CD47 overexpression under hypoxia [82]. Moreover, both innate and adaptive immunity are negatively impacted by the CD47-SIRP axis. The CD47-SIRP axis has proven to be a valuable immunotherapy target in cancer, and the use of anti-CD47 antibodies in various solid tumors is now being studied [80].

### VISTA

An investigation conducted recently showed that the hypoxic TME of colorectal cancer (CRC) mouse models and humans overexpresses VISTA, a B7 family negative checkpoint modulator [83]. When HIF-1α attaches to the HRE in the VISTA promoter, myeloid cells, such as DCs, macrophages, and MDSCs, selectively express VISTA in the hypoxic TME [84]. VISTA expression brought on by hypoxia can inhibit T cell function and proliferation [83, 85]. Additionally, hypoxic lymphocytes have elevated levels of co-stimulatory and co-inhibitory receptors like OX40, CTLA-4, CD137, and LAG-3 than lymphocytes under normoxia environments [86]. This association between HIF-1 and these higher levels of co-inhibitory and co-stimulatory receptors has been demonstrated.

Drugs that disrupt these pathways are now used to treat a wide range of malignancies and exhibited sustained therapeutic effects in some cancer patients. The next generation of immune checkpoints tends to function in synergy with chemotherapy or other CPIs due to its distinct action mechanism in comparison to previous anticancer methods.

### CD73

CD73 expression in the tumor microenvironment has been investigated as a predictive biomarker for clinical outcomes in a variety of tumor types, with a statistically significant correlation between high CD73 expression and poor clinical results [87]. This is consistent with adenosine’s activity as an immunosuppressive metabolite [88]. The overexpression of CD73/A2aR is frequently attributed to genetic variations [89], which in turn leads to immunosuppression by modulating the tumor microenvironment [90–92]. HIF-1α accumulation has been shown to promote downstream CD73 overexpression, activating the CD73-adenosine pathway and reducing T cell effector activity [93]. In the latest study, Yuan et al. created cancer cell membrane-camouflaged gelatin nanoparticles (CSG@B16F10) to distribute oxygen-producing molecules catalase and CD73siRNA simultaneously, improving tumor oxygenation and reducing CD73-adenosine pathway-mediated T cell immunosuppression [94]. An appealing and potential target in cancer immunotherapy is the immunosuppressive effect of hypoxia signaling on NK cells via the HIF-dependent CD73-adenosinergic pathway. In the context of solid tumors, the administration of drugs that can block CD73 and/or target HIFs in addition to NK cell-based therapies is becoming recognized as an immunotherapeutic approach with substantial promise [95].

In hypoxic tumor cells such as MDSCs, macrophages, and DCs HIF-1 stabilization promotes the overexpression of PD-L1. The increased level of CD47 on the tumor cell surface is attributed to HIF-1. Powerful “don’t eat me” signals are sent to cancer cells when CD47 binds to SIRP, which is extensively upregulated on myeloid-linage hematopoietic cells including TAMs and MDSCs thus preventing phagocytosis. The tumor cell surface expresses a great number of HLA-G when exposed to hypoxia. The overexpressed HLA-G attaches to its inhibitory receptors on immune cells, inducing immunosuppression and facilitating immunological evasion by compromising the DC antigen presentation, activation of suppressor T-cells, and inhibition of cytotoxic attack. Hypoxia may induce VISTA overexpression on myeloid cells such as macrophages, MDSCs, and DCs once HIF-1 interfaces with the HRE in the VISTA promoter, which inhibits the activity and proliferation of T cells. The expression of co-stimulatory factors (OX40 and CD137) and inhibitory immune checkpoints (CTLA4 and LAG-3) on the surface of T cells rises in response to hypoxia. A hypoxic environment inhibits the levels of co-stimulatory molecules such as CD86, CD80, and CD40 on DCs, via the mechanism of stabilizing HIF-1.

## HIF activators and inhibitors

### HIF agonists

**Fig. 5:**
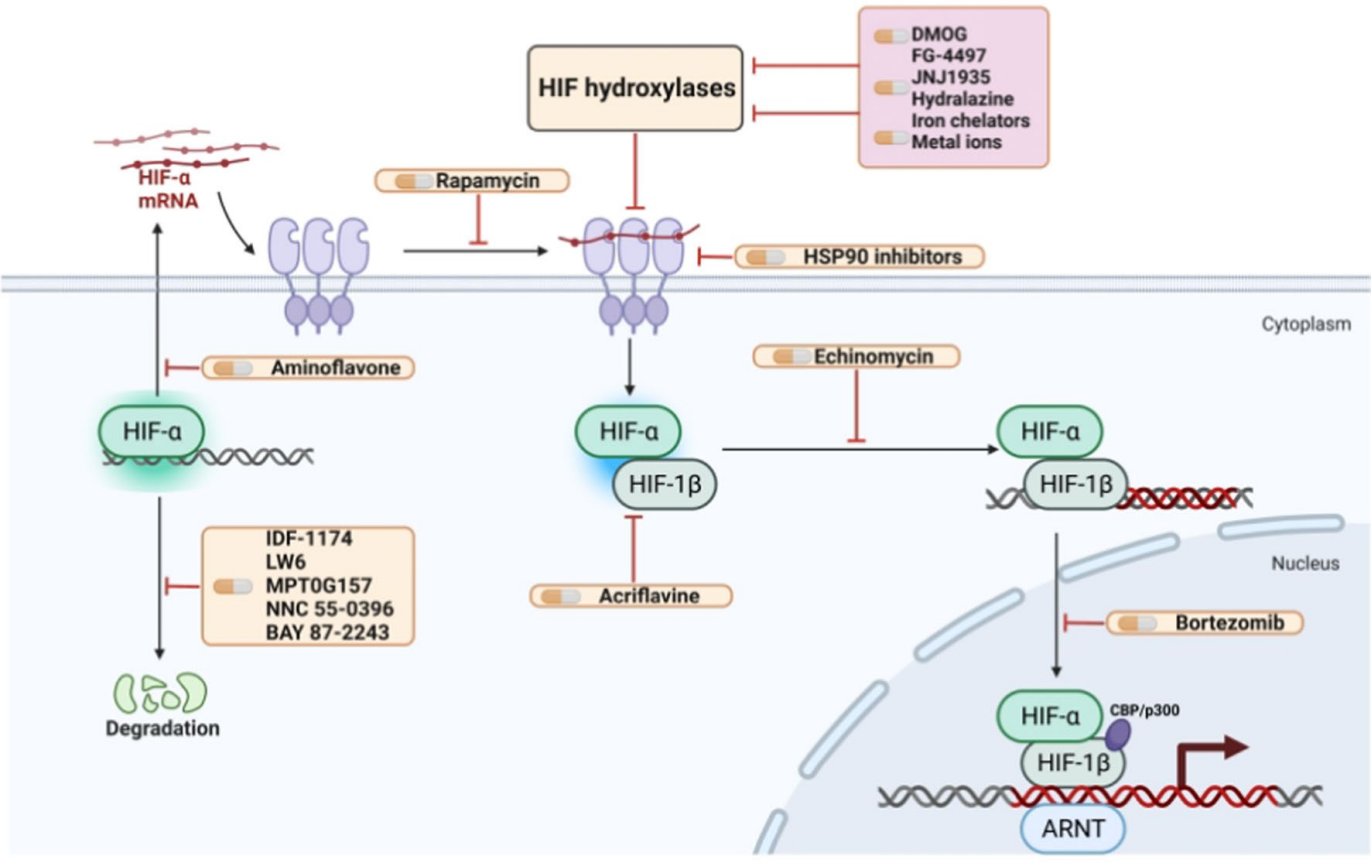
HIF pathway-targeting pharmaceuticals

Several pharmaceutical therapies that stimulate the HIF pathway are presently identified, particularly hydroxylase inhibitors such as dimethyloxalylglycine (DMOG), a 2-oxoglutarate mimic that stimulates the expression and activity of HIF in vivo and in vitro [96]. Several other similar hydroxylase inhibitors, such as JNJ1935 and FG-4497, have also been identified [97, 98]. Upon treatment with A-503451A, the expression of erythropoietin, vascular endothelial growth factor, and HIF-dependent genes, were increased at the protein and mRNA levels [99]. Potent HIF activators include iron chelators like desferrioxamine and metals like cobalt [100]. Even though the exact action mechanism of the cardiovascular medication hydralazine, which has been employed to treat cardiovascular diseases for many years, is still unknown, it has been demonstrated to have a potent hydroxylase inhibitory effect [101]. As a result, multiple examples of possible HIF-activating drugs have been presented, as shown in Fig. 5.

### HIF inhibitors

The de-repression in hypoxia-based regulation of the HIFs system makes it more challenging to develop HIF inhibitors rationally. Although the particular processes by which many of these medications directly repress HIFs are still largely unknown, a range of potential HIF inhibitors has been discovered through several screening techniques, many of which included evaluating huge libraries of substances against simple assays of HIF activity. Importantly, HIF suppression is a topic of ongoing research in cancer treatment [6, 102]. There have been reports of HIF inhibitors, such as medications that interfere with a variety of processes, like HIF-1α mRNA expression (e.g. aminoflavone [103]), HIF-1α protein synthesis (e.g. rapamycin [104]), HIF-1α protein stabilization (e.g. HSP90 inhibitors [105]), HIF heterodimerization (e.g. acriflavine [8]), HIF-DNA binding (e.g. echinomycin [11]) and HIF transactivation (e.g. bortezomib [106, 107]). Table 1 presents possible action mechanisms of a few HIF inhibitors (not exhaustive).

**Table 1:** HIF inhibitors along with their respective action mechanisms

Mechanism	Compound	References
HIF mRNA production	Aminoflavone	[103]
HIF protein synthesis	Rapamycin	[104]
	Apigenin	[114]
	Temsirolimus (CCI-779)	[114]
	Everolimus (RAD-001)	[114]
	Digoxin	[115]
	2-meth-oxyestradiol, taxotere	[116]
	Topotecan	[117]
	NSC-644221	[118]
	EZN-2968	[119]
	YC-1	[120]
	PX-478	[121]
	EZN-2208	[122]
	Glyceollins	[123]
	VEGFR inhibitors	[124]
	Tempol	[125]
HIF protein stabilization	HSP90 inhibitors	[115]
	Antioxidants	[126]
	BIX01294	[127]
	PX-12	[128]
	LAQ824	[129]
	G-rich oligonucleotides	[130]
	Berberine	[131]
	Se-methylselenocysteine	[132]
	YC-1	[133]
HIF dimerization	Acriflavine	[8]
	PT2385	[134]
	PT2399	[135]
	Belzutifan	[136]
HIF degradation	IDF-1174	[137]
	LW6	[138]
	MPT0G157	[139]
	NNC 55-0396	[140]
	BAY 87-2243	[141]
HIF subunit heterodimerization	Acriflavin	[69]
	CCS 1477	[142]
HIF-DNA binding	Echinomycin	[11]
	Anthracyclines	[143]
HIF transactivation	Bortezomib	[106, 107]
	Belinostat, chidamide, pabinostat, romidepsin, vorinostat	[144]

Recently, efforts have been undertaken to create targeted HIF-2 inhibitors that are presently being tested in clinical trials [108]. Among the many clinical concerns in the context of cancer, chronic hypoxia is one of them, and the prospect of HIF-2-directed therapeutics is now feasible, judging by recent findings that identify a key HIF-2 target gene, namely, tubulin beta-3 chain (TUBB3), which is involved in tumor advancement and chemotherapy [109]. Conversely, diseases characterized by ischemic/hypoxic states and inflammatory disorders might both be treated by stimulating the protective HIF response. The principal targets of the several compounds suggested to trigger HIF signaling are PHD2, PHD3, and VHL. In terms of clinical development, PHD3 inhibitors are the most advanced [110–112]. Nevertheless, current research suggests that in addition to regulating the HIF response, FIH1 and PHDs also modulate a variety of other cellular metabolic processes [113], which requires further consideration to reduce any potential negative outcomes.

Multiple HIF inhibitors (yellow boxes) and HIF activators (purple boxes) drugs identified to date. Among them, HIF inhibitors interfere with a variety of processes, such as Hif-α mRNA expression, Hif-α protein synthesis, Hif-α protein stabilization, HIF heterodimerization, HIF-DNA binding, and Hif-α transcriptional activation.

## Several HIF inhibitors in malignant tumors

A few biological processes that are regulated by hypoxia-dependent HIF-1 and HIF-2 encompass angiogenesis, EMT, proliferation/survival of tumor cells, the cancer stem cells (CSCs) maintenance, metastasis/invasion, metabolic reprogramming, and immunomodulation [145, 146]. Multiple HIF inhibitors are now being researched in pre-clinical and clinical studies since HIF performs a significant function in cancer, and this pathway presents a possible treatment target [147, 148]. Several compounds are now undergoing phase II clinical studies, either alone or in conjunction with available anticancer medications, mostly to treat advanced or resistant malignancies [148]. Several approaches have been put forth to combat hypoxia in cancer, including the use of hypoxia-activated prodrugs (HAPs), suppression of HIF signaling, metabolic intervention, as well as downstream targeting of crucial hypoxic pathways, such as the mTOR and unfolded protein response (UPR) pathways; others are still in the conceptual design phase, such as siRNA-mediated gene treatments and recombinant anaerobic bacteria [149].

Notably, Belzutifan (MK-6482), a specific small molecule inhibitor that targets the TF HIF-2, was shown to shrink tumors and stop tumor growth in over 90% of VHL patients in a 3-year research [150]. Belzutifan recently received FDA approval to treat adult patients with pancreatic neuroendocrine tumors, central nervous system hemangioblastomas, and VHL-associated ccRCC [151]. Belzutifan's effectiveness is now being assessed in multiple other solid tumors, such as glioblastoma (NCT02974738). Furthermore, several clinical trials are currently conducted involving patients with ccRCC evaluating combinatorial treatment approaches including immune-checkpoint therapies or anti-angiogenic therapies used in conjunction with belzutifan, like Belzutifan + Pembrolizumab (PD-1 inhibitor) + Lenvatinib (NCT04736706), Belzutifan + Cabozantinib (TKI) (NCT03634540), and Belzutifan + Lenvatinib (TKI) (NCT04586231) [152–154].

The current study discovered that removing stromal hypoxia-inducible factor-2 from animals with pancreatic cancer reduced tumor development and increased median survival, potentially by interfering with immunosuppressive cancer-associated fibroblast-macrophage interaction [155]. PX-478, a HIF-1α inhibitor that could also inhibit HIF-2α, slows the growth of pancreatic ductal adenocarcinoma and esophageal squamous cell cancer both in in vivo and in vitro settings [156, 157]. HIF-1α production and activation are inhibited at various levels by bortezomib because it prevents the recruitment of p300 and hinders the PI3K/Akt/TOR pathway, without affecting HIF-2α [107, 158]. In a similar vein, a range of anti-cancer medications, including those that block the PI-3 K-mTOR pathway, histone deacetylases (HDAC), or topoisomerase, exhibit indirect impacts and varying degrees of effectiveness in reducing the production of and activation of HIF-1/2α [159].

Salman et al. reported the development of 32-134D, a low-molecular-weight compound that suppressed the expression of genes regulated by HIF-1/2 in cancerous cells and prevented tumor growth [160]. The low-molecular-weight drug was developed to specifically target HIF-1/HIF-2, resulting in the degradation of the HIF- component and blocking transcription of HIF-1/2 target genes. This suppression influenced genes involved in glycolysis, angiogenesis, and immune regulation. Furthermore, mice with tumors developed fewer tumors following HIF inhibitor administration.

## HIF inhibitors combined with conventional chemoradiotherapy

Hypoxia contributes to the failure of conventional cancer therapies such as chemotherapy [161] and radiotherapy [162]. Observations from multiple murine models show that therapies with VEGF receptor inhibitors, such as the small molecule tyrosine kinase inhibitor sunitinib or the anti-VEGFR2 antibody DC-101, decreased the vascularization and growth of primary tumors but accelerated metastasis, likely since deficient angiogenesis contributed to enhanced intratumoral hypoxia as well as increased HIF activity [163–165]. The FDA revoked the clearance of the anti-VEGF antibody bevacizumab because it did not affect the advancement of breast cancer [166] which may entail additional angiogenic growth factors being expressed in a HIF-1-dependent way. Conversely, HIF inhibitor remarkably alleviated the spontaneous metastasis of human breast cancer cells to the lungs of mice orthotopic transplant models by interfering with several metastatic process phases [20, 167]. Numerous studies also show that HIFs have a role in radiotherapy [22] and chemotherapy [23] resistance and increasing data suggest that HIF-1 activity could perform a role in the development of resistance to new targeted medicines, including imatinib therapy for chronic myeloid leukemia [168]. Together, these findings imply that the co-administration of HIF inhibitors can increase the effectiveness of antiangiogenic drugs, and mouse model studies corroborate this hypothesis [169]. When conventional chemotherapy is used in conjunction with a HIF inhibitor, it also might be more successful; this effect is underlied by a variety of molecular pathways that depend on the cell type and are specific to chemotherapy.

## Cross-talk between HIF and tumor immunotherapy

The treatment environment for human tumors in advanced clinical stages has significantly changed over the past several decades, thanks to immunotherapy, a ground-breaking intervention [170]. Adoptive cell transfer (ACT) and CPIs are two examples of immunotherapeutic interventions that use the components of the immune system to combat malignant cells [171]. Unlike conventional treatment approaches, CPIs function by reactivating the host immunity to combat malignant cells. A balanced state is maintained between pro- and anti-inflammatory signals by immune checkpoints [172]. These immune checkpoints encompass a set of stimulating and inhibiting mechanisms responsible for regulating the activity of immune cells [173]. Evidently, hypoxia could be thought of as a possible target in combination with cancer immunotherapy due to HIF’s crucial function in controlling tumor development and immune surveillance. In this part, we investigate the prospect of altering hypoxia to enhance the success of cancer immunotherapies using data from pre-clinical and clinical research.

### HIFs associated with immune checkpoint blockade

The significance of the immune checkpoint in immune tolerance vs. tumor escape of host immune response defines the proportionate risk/benefit ratio [174]. The predominantly applied immunotherapy interventions in the past decade are antibodies against immune inhibition receptors including PD-L, PD-1, and CTLA-4 [175]. The US FDA has licensed three separate classes of CPIs to treat distinct cancers, namely CTLA-4 inhibitors (Ipilimumab), PDL-1 inhibitors (Avelumab, Durvalumab, and Atezolimumab), and PD-1 inhibitors (Cemiplimab, Pembrolizumab, and Nivolumab) [176]. Immune tolerance enhancement by differential PD-L1 regulation in normal and malignant tissues is an essential method for safer and more effective immunotherapy. In some cancer patients, PD-1/PD-L1 inhibitors can produce positive therapeutic outcomes. PD-1/PD-L1 inhibition-based combination therapies are available for a majority of cancer subtypes and may prolong patient survival [177]. Approximately 50% of individuals with cancer are candidates for ICI immune checkpoint inhibitor (ICI) therapy, and a large proportion of patients acquire sustained responses [178, 179]. Nonetheless, only a portion (20–40%) of patients respond well to this treatment, emphasizing the rising demand for the development of predictive biological markers [180]. Preliminary clinical trials of the anti-CTLA-4 antibody ipilimumab showed long-lasting responses and significant survival benefits in certain melanoma patients [181]. Patients who have advanced and otherwise incurable melanoma, NSCLC, and urothelial tumors responded similarly to PD-1 and PD-L1 blockade [182, 183]. Conversely, high-grade immune-related adverse events (irAEs), such as hepatitis, pneumonitis, thyroiditis, dermatitis, and colitis were linked to both CTLA-4 inhibition and PD-1/PD-L1 blocking [184–186]. These iAEs were most likely caused by the failure of self-tolerance caused by ICI [187, 188]. Combinatorial therapeutic targeting was made possible thanks to the complementing immunosuppressive properties of CTLA-4 and PD-1. Patients with RCC and melanoma attained an improved response rate after the administration of the ipilimumab + nivolumab (anti-PD-1) combination scheme [189, 190], even though it means experiencing higher-grade irAEs more frequently [186]. In the meantime, the practical usefulness of CPIs is hampered by toxicity associated with the therapy as well as tumor-specific or acquired tolerance to CPIs [191].

It has been demonstrated that hypoxia alters the levels of immune checkpoints like CTLA-4, PD-1/L-1, CD47, and TIM3 to modify the immune cell-induced anti-tumor response, thus suppressing immune surveillance [64]. Moreover, CD137 (4-1BB) is a member of the TNF receptor family that was first discovered on activated T lymphocytes. CD137 expression is greatly preferred in TILs as a result of a hypoxia-dependent HIF-1 response. Low doses of anti-CD137 mAb targeted TILs given within tumors have been proven in studies to have systemic therapeutic benefits and to function synergistically with systemic inhibition of the PD-1/B7-H1 (PD-L1) pathway [86]. HIFs targeting in immunotherapy is a fairly novel idea, with ample evidence of its viability provided by others [72, 73, 192]. Furthermore, a handful of reports have shown that CPIs coupled with various treatment modalities, including chemotherapy [193], radiation treatment, HIF inhibitors, and cancer vaccines can successfully overcome tumor resistance to ICI therapy.

### Combination therapy of CPIs and HIF inhibitors

When the HIF-1α/PD-L1 axis in malignant cells is targeted, it leads to tumor rejection and the reactivation of tumor-infiltrating lymphocytes (TILs). For example, in vivo administration of PX-478 and anti-PD-1 antibodies inhibits tumor development and lengthens animal life, which is linked to decreased suppression of immunity, increased TIL homing, and decreased expression of EMT phenotypes [194]. Inhibiting the transcriptional activities of HIF-1α increases NK cells and CTLs mediated by CCL2- and CCL5 in the tumor bed in a murine melanoma model, thus enhancing the anticancer effects of peptide vaccine and anti-PD-1 blocking antibodies [69]. One study illustrated that HIF-1 suppression promoted PD-L1 overexpression in healthy regions while blocking PD-L1 in the tumor site [195]. According to Zandberg et al., mice with intra-tumoral hypoxia exhibited modified disease control rate (DCR) and survival in an animal model of HNSCC, making them non-responsive to anti-PD-1 rituximab [196]. Importantly, the proportion of mice that responded completely to anti-PD-1 CPI rose from 25 to 67% following treatment with the HIF inhibitor [160].

In preclinical studies, the topoisomerase 1 and HIF-1α dual inhibitor CRLX101 also displayed positive synergy with immunotherapeutic regimens [58]. Combining ICI (anti-CTLA-4/PD-1) with HIF-1-mediated ectoenzyme ENTPD2 inhibitors remarkably increased the levels of TILs and prolonged the lifespan of mice with tumors relative to ICI treatment alone [66]. Notably, hypoxia-triggered HIF pathways comprise a sophisticated network of several intersecting cascades, and the utilization of combo treatment calls for more research. Hence, combining HIF inhibitors and CPIs can be promising for increasing anticancer immune response while decreasing tumor cell plasticity and therapeutic resistance [197]. These results offer what we consider to be a novel approach to the development of immunotherapeutic drugs. HIF suppression could therefore be a useful ally for different CPIs.

### HIF inhibitors decreased irAEs

Since CPIs operate to activate T-cell responses, it is not unexpected that these compounds might result in irAEs, some of which can be severe or perhaps deadly [187, 198]. The possible severity and mortality of ICI-related toxicities are becoming more well-recognized [198, 199]. Fatalities appear to occur randomly and often early following the initiation of therapy [198]. The prevention of autoimmune conditions may be aided by CTLA-4, a repressive factor controlling T-cell immune reactions. Nevertheless, blocking it with ipilimumab could result in irAEs like enterocolitis and colitis [200]. Correspondingly, monoclonal antibodies targeting PD-1 and PD-L1 are not as toxic as those against CTLA-4 [190]. Unfortunately, the present method, which resolves tumor escape from host defense, also impairs the immune tolerance checkpoint, resulting in severe irAEs, especially when combined with anti-CTLA-4 antibodies. Notably, the severity of fatal organ involvement varies across anti-CTLA-4 and anti-PD-1/PD-L1 interventions, with colitis being the most commonly observed in the former and neurotoxicity, hepatitis, and pneumonitis being recorded in the latter. [201–203]. Myocarditis and colitis are the most prevalent causes of death resulting from combination therapy (e.g., anti-PD-1/CTLA-4) [198].

According to the most current research data, the HIF-1 inhibitor echinomycin enhanced the anti-CTLA-4 treatment’s cancer immunotherapy effectiveness, with efficacy comparable to anti-CTLA-4 + anti-PD-1 antibodies [195]. Bailey et al. claim that inhibiting HIF-1 raises the immune tolerance checkpoint in healthy cells in addition to preventing immune escape in the TME [195]. Therefore, eliminating irAEs while maintaining the synergistic benefits of dual ICB is a key challenge for cancer immunotherapy.

## Discussions and perspectives

Identifying small chemical inhibitors that precisely target the HIF pathway has been attempted extensively to date (reviewed in [204]). Nonetheless, investigations of HIF-1 activities in cultured tumor cell lines are the basis for the majority of HIF inhibitors that have been found thus far. None of the inhibitors that are currently on the market appear to have the HIF-1 pathway as their only target [205]. Additionally, little is documented about their selectivity for other HIF-subunits (HIF-2/3 isoforms) and their capacity to influence the HIF switch. Currently, there is no medication available that only targets HIFs without interfering with other pathways. HIF inhibition or activation may have negative effects on living things, predicated on the therapeutic setting. Due to the potentially detrimental consequences of disrupting the HIF pathway, whether positively or negatively, developing strategies to precisely convey these medications to the targeted site while minimizing systemic exposure is an essential therapeutic consideration when exploring the use of HIF modulators.

Drug resistance is still a significant concern in anticancer therapy, and it is anticipated that HIF-1 inhibitors would have the same difficulty. Therefore, it must be determined if the on-specific effects of HIF inhibition are helpful for polypharmacy or harmful in terms of side effects. Multiple preclinical models of HIF-1-deficient tumors exhibit initially slow growth, followed by rapid growth, suggesting the presence of resistance and evasion mechanisms from HIF-1 inhibition [206–209]. By inhibiting tumor hypoxia, anti-hypoxia therapy reduces the expression of CD39 on the most terminally exhausted T cells, limiting its regulatory potential and promoting the improvement of CD8 + T cell effector function [210]. An increasing amount of evidence suggests that immunotherapy targeting immune checkpoints may be of greater significance after alleviating tumor hypoxia. It is crucial to comprehend the HIF-1-independent processes that may control these metabolic alterations in hypoxic cancerous cells to prevent the possible challenge of resistance to treatment.

The impressive anticancer effects for the majority of combinations are mostly restricted to animal tumor models. Choosing the best preclinical model to determine the activities of combination regimens is a major difficulty. Overall, compared to syngeneic murine models, which are often used, humanized models developed from patients may provide a more useful assessment of efficacy.This is undoubtedly the case for both combination immunotherapy and HIF inhibitor-based clinical anti-cancer treatment. A difficult task in the development of combination treatment is optimizing the delivery strategy, which includes dose, scheduling, and sequencing. Choosing the ideal medicine combination and finding the biomarkers that show treatment efficacy are other unsolved problems. Due to the heterogeneity and progression of tumors, a liquid biopsy could provide a real-time biological marker for guiding personalized immunotherapy by dynamically monitoring the immune milieu of the TME [211]. In summary, we believe that patients should get a customized mix of treatments based on immunological profile and other prognostic factors and HIF inhibitors may eventually offer better clinical cancer therapy options.

## Data Availability

Not applicable.

## Abbreviations

TFs: Transcription factors
HIFs: Hypoxia-inducible factors
CPIs: Immune checkpoint inhibitors
O_2_: Oxygen
TFs: Transcription factors
EMT: Epithelial-mesenchymal transition
VHL: Von Hippel-Lindau
ARNT: Aryl hydrocarbon receptor nuclear translocator
PAS: PER-ARNT-SIM
bHLH: Basic helix-loop-helix
TADs: Transactivation domains
ODD: Oxygen-dependent degradation domain
FIH-1: Factor-inhibiting hypoxia-inducible factor-1
PHDs: Prolinehydroxylases
pVHL: VHL tumour suppressor protein
Fe^2+^: Iron ions
CBP/CREBBP: CREB-binding protein
HRE: Hypoxia response element
SIRT1: Sirtuin 1
TME: Tumor microenvironment
MDSCs: Myeloid-derived suppressor cells
TAMs: Tumor-associated macrophages
MICA: Major histocompatibility complex class I chain-related molecule
HCC: Hepatocellular carcinoma
ENTPD2: Ectonucleoside triphosphate diphosphohydrolase 2
ICB: Immune checkpoint blockade
SOD3: Superoxide dismutase
EC: Endothelial cells
IFN: Interferon
VISTA: V-domain Ig suppressor of T-cell activation
CD47: Cluster of differentiation 47
HLA-G: Human leukocyte antigen G
ccRCC: Clear cell renal cell carcinoma
CAIX: Carbonic anhydrase 9
CRC: Colorectal cancer
DMOG: Dimethyloxalylglycine
TUBB3: Tubulin beta-3 chain
CSCs: Cancer stem cells
HAPs: Hypoxia-activated prodrugs
UPR: Unfolded protein response
HDAC: Histone deacetylases
ACT: Adoptive cell transfer
ICI: Immune checkpoint inhibitor
irAEs: Immune-related adverse events
TILs: Tumor-infiltrating lymphocytes
